# Thoracoabdominal Normothermic Regional Perfusion: Real-world Experience and Outcomes of DCD Liver Transplantation

**DOI:** 10.1097/TXD.0000000000001767

**Published:** 2025-02-28

**Authors:** Yanik J. Bababekov, Anna H. Ha, Trevor L. Nydam, Carlos Goncalves, Rashikh Choudhury, JoLynn Shinsako, Maria Baimas-George, David M. Reynolds, Cassidy Yoshida, Caroline A. Racke, Han Grewal, Sophia Pomposelli, Ivan E. Rodriguez, Jordan R.H. Hoffman, Jesse D. Schold, Bruce Kaplan, Elizabeth A. Pomfret, James J. Pomposelli

**Affiliations:** 1 Department of Surgery, University of Colorado Anschutz Medical Campus, Aurora, CO.; 2 Colorado Center for Transplantation Care, Research and Education (CCTCARE), Aurora, CO.

## Abstract

**Background.:**

Donation after circulatory death liver transplantation (DCD LT) is underused given historical outcomes fraught with ischemic cholangiopathy (IC). We aimed to assess 6-mo IC in LT from DCD via normothermic regional perfusion (NRP) compared with DCD via static cold storage (SCS).

**Methods.:**

A retrospective review of adult Maastricht-III DCD liver donors and recipients at the University of Colorado Hospital from January 1, 2017, to August 27, 2024, was performed. The 6-mo IC rate was compared between NRP and SCS. Secondary outcomes included biochemical assessments of accepted versus declined NRP liver allografts and allograft and patient survival for NRP and SCS groups.

**Results.:**

One hundred sixty-two DCD LTs (SCS = 79; NRP = 97) were performed and 150 recipients (SCS = 74; NRP = 86) reached 6-mo follow-up. Six-month IC was lower for NRP compared with SCS (1.2% versus 9.5%, *P* = 0.03). The Donor Risk Index (2.44 [2.02–2.82] versus 2.17 [1.97–2.30], *P* = 0.002) and UK DCD Risk Score (4.2 ± 2.9 versus 3.2 ± 2.3, *P* = 0.008) were higher for NRP versus SCS. The Liver Graft assessment Following Transplantation score was less for NRP compared with SCS (–3.3 versus –3.1, *P* < 0.05). There were several differences in median biochemical parameters during NRP between accepted and declined livers, including higher terminal biliary bicarbonate (22.7 [20.9–29.1] versus 10.8 [7.6–13.1] mEq/L, *P* = 0.004). There were no significant differences in 12-mo allograft or patient survival for NRP versus SCS.

**Conclusions.:**

NRP is a disruptive innovation that improves the utilization of DCD livers. Despite higher-risk donor-recipient pairing for NRP compared with SCS, we demonstrate a decrease in IC for NRP. These data facilitate benchmarking of thoracoabdominal NRP DCD LT and support further protocol development.

The severe organ shortage in the United States justifies the expansion of the donor pool via donation after circulatory death (DCD). Annually, the number of additions to the liver transplant (LT) waitlist outpaces transplants performed, and annual waitlist mortality is about 20%.^1^ Although accepting a DCD offer is associated with a 46% reduction in mortality compared with staying on the waitlist, DCD LT is underused.^2-4^ Some US programs remain cautious about DCD LT because of historical reports of increased risk for early allograft dysfunction (EAD), ischemic cholangiopathy (IC), and poor patient outcomes.^5-8^

Abdominal and thoracoabdominal normothermic regional perfusion (A-NRP and TA-NRP) procurement for DCD donors has been implemented in the United States with recently established standards by the American Society of Transplant Surgeons (ASTS).^9-12^ Early data suggest potential for acceptable outcomes.^13-15^ TA-NRP has the advantage of increasing organ utilization per donor by including perfusion of thoracic organs.^16^

Although NRP is relatively new in the United States, some European countries have been applying A-NRP for DCDs during the past decade giving excellent results compared with the standard rapid recovery of DCDs with static cold storage (SCS).^17-21^ Spain has followed a national A-NRP protocol since 2012 and has demonstrated decreased biliary complications, ischemic-type biliary lesions, graft loss, and mortality compared with SCS.^19^ Moreover, a UK report indicated that A-NRP is associated with a decreased risk of biliary complications compared with SCS and normothermic machine perfusion (NMP).^22^ DCD-NRP is expanding in more European countries such as Sweden^23^ and is being used in other countries for extended criteria DCDs.^24-28^

US data are emerging with limited conclusions secondary to unclear utilization rates, relatively lower-risk donors and recipients, and heterogeneous NRP procurement protocols.^14,29-33^ We describe the largest known single-center experience using TA-NRP for DCD LT and assess NRP’s impact on 6-mo IC compared with DCD LT via SCS.

## MATERIALS AND METHODS

### Study Design

A retrospective review of all potential Maastricht-III (controlled donation in a person awaiting circulatory arrest) DCD liver donors and their recipients aged 18 y or older at the University of Colorado Hospital (UCH) from January 1, 2017, to August 27, 2024, was performed. We excluded DCD allografts if transplanted via NMP or sequential NRP-NMP. Hypothermic oxygenated machine perfusion was not available for clinical use during the study period. The Colorado Multiple Institutional Review Board (protocol No. 22-2002) approved the study.

### Data Collection

Data from DCD liver offers that UCH accepted with intent to procure were obtained from United Network for Organ Sharing DonorNet. Periprocurement clinical and laboratory information during NRP was collected using the Colorado Organ Assessment Form (**Figure S1, SDC,** http://links.lww.com/TXD/A740). DCD LT recipient information was extracted from the electronic medical record. The model for end-stage liver disease (MELD)-sodium score, Donor Risk Index (DRI), UK DCD Risk Score, Distressed Community Index, and grade according to Clavien-Dindo Classification were calculated.^34-38^ Allocation MELD was obtained from DonorNet at the time of liver offer. Data regarding organ yield during the study period for all DCD and DBD donors in our donor service area were obtained from the local organ procurement organization (OPO). Precise hepatectomy times during DCD procurement are not available for review; however, UCH surgeons routinely aim for <30 min. For each potential DCD case, premortem heparin was administered, but premortem cannulation was not performed.

The primary outcome, IC within 6 mo from transplant, was defined as a clinically significant nonanastomotic stricture of the biliary tree with patent vasculature. Our center does not follow a strict surveillance protocol for IC. IC was diagnosed by either endoscopic retrograde or magnetic resonance cholangiopancreatography given clinical suspicion during follow-up. Secondary outcomes included differences in biochemical parameters during NRP between accepted and declined livers, primary nonfunction (PNF) defined by United Network for Organ Sharing criteria,^39^ EAD by Olthoff criteria,^5^ Liver Graft assessment Following Transplantation (L-GrAFT) score,^40^ Model for Early Allograft Function Scoring (MEAF),^41^ biliary and vascular complications, and surrogates for resource utilization including transfusion requirements, postoperative disposition, need for renal replacement therapy, length of stay, and readmission.

### DCD With SCS Recovery Practice

In 2017, UCH began to use tissue plasminogen activator (tPA) for DCD LT to minimize biliary complications.^42^ We administered 100 mg of tPA in 1 L of normal saline in the first liter of aortic flush at procurement, followed by 4–6 L and 2–4 L of preservation solution in the arterial and portal venous systems, respectively. We also administered 0.1 mg/kg donor weight of tPA into the allograft hepatic artery after reperfusion of the portal vein in the recipient. Our center followed the Organ Procurement and Transplantation Network (OPTN) definition of functional warm ischemia time (fWIT) of systolic blood pressure of <80 mm Hg or oxygen saturation of <80% and avoided allografts with fWIT >30 min. Donor-recipient matching was guided by the UK DCD Risk Score, and we aimed for a score of ≤5 (low risk).

### DCD With NRP Recovery Practice

We began accepting NRP DCD livers in February 2022 and performed our first TA-NRP case in October 2022. UCH cardiothoracic transplant surgeons trained the UCH abdominal transplant surgeons to perform TA cannulation to facilitate the pursuit of abdominal-only DCD offers. Either TA-NRP or A-NRP was performed by abdominal surgeons if thoracic organs were declined a priori. If thoracic organs were in consideration at the time of procurement, then NRP was performed by the thoracic team. All organs were recovered from the donor after aortic cross-clamp and cold flush. A-NRP cases were only performed by abdominal procurement surgeons when thoracic organs were declined a priori and thoracic anatomy made TA-NRP not feasible.

We followed a preprocedure huddle per ASTS standards.^11^ Our DCD TA-NRP technique was previously described,^43^ and our NRP practice followed technical and ethical guidelines as described in the ASTS standards.^9-12^ In accordance with ASTS standards, the brachiocephalic vessels were controlled in TA-NRP such that cerebral flow would be prevented by a combination of suture ligature, clamping, and venting when appropriate. In A-NRP, the descending thoracic aorta was controlled with a combination of a clamp and suture ligature. In both TA-NRP and A-NRP, circuit flow confined to the intended cavity of perfusion was confirmed by the absence of flow on peripheral arterial lines. We sent an institutional NRP procurement team to every DCD offer from our OPO and did not have strict institutional criteria to decline an NRP allograft a priori. The UCH NRP team included perfusionists, a perfusion pump (Spectrum Medical, Fort Mill, SC), and point-of-care laboratory testing (Piccolo and i-STAT devices, Abbot Rapid Diagnostics Informatics Inc, Charlottesville, VA). fWIT was defined by OPTN criteria. Unlike DCD LT with SCS, we would accept fWIT up to 45 min with NRP. For abdominal-only organ offers, TA-NRP was preferred over A-NRP as we initially learned the technique from cardiothoracic surgeons. SCS was used when NRP was not available.

NRP circuit parameters were managed by a perfusionist and included maintaining normothermia and blood flow rate per body surface area of ≥2.2 L/min/m^2^ or goal-directed perfusion maintaining oxygen delivery index of ≥280 mL/min/m^2^_._ Viability assessment of the liver was performed for up to 120 min and recovery of the abdominal organs occurred in s standard manner. Information for allograft assessment was collected on an institutional form (**Figure S2, SDC,** http://links.lww.com/TXD/A740). Biochemical assessment included laboratory draws about every 15 min and assessed perfusion, hepatocellular, and cholangiocellular function.^26^ We relied on macroscopic inspection and a biopsy if requested by the on-call surgeon.

Bile was collected via a closed system using a biliary catheter placed into the distal common bile duct and then analyzed with an i-Stat as described in prior reports.^26^ Bile was collected at the start of NRP for a baseline assessment and then in 15-min intervals as clinically feasible. The terminal bile sample was obtained as part of the clinical review just before the decision of organ acceptance at the end of the NRP run. We followed prior observations to accept livers with the relative stability of transaminases, decreasing trend in lactate, alkaline bile pH, and acceptable macroscopic appearance.^19,21,26,33^

### Statistical Analysis

Descriptive statistics of categorical and continuous variables were expressed as count (percentage) and median (interquartile range), respectively. Univariable analyses were performed using the Fisher exact test and Wilcoxon rank-sum test for categorical and continuous variables, respectively. IC at 6 mo was assessed via the Fisher exact test. Kaplan-Meier survival curve estimates of patient survival and graft survival noncensored for death and censored for death were compared using the log‐rank test. Graft survival was defined as the time from transplant to graft failure. Organ yield was calculated by assessing the means of organs procured for transplant from each donor type. Because of the paucity of pancreas and intestine transplants during the study period, yields of these organs were excluded. Outcomes were compared between SCS versus NRP with statistical significance set at a *P* value of <0.05. Analyses were undertaken using Python version 3.11.5.

## RESULTS

### Institutional DCD LT Offers and Transplant Volume: SCS vs NRP

**FIGURE 1.**
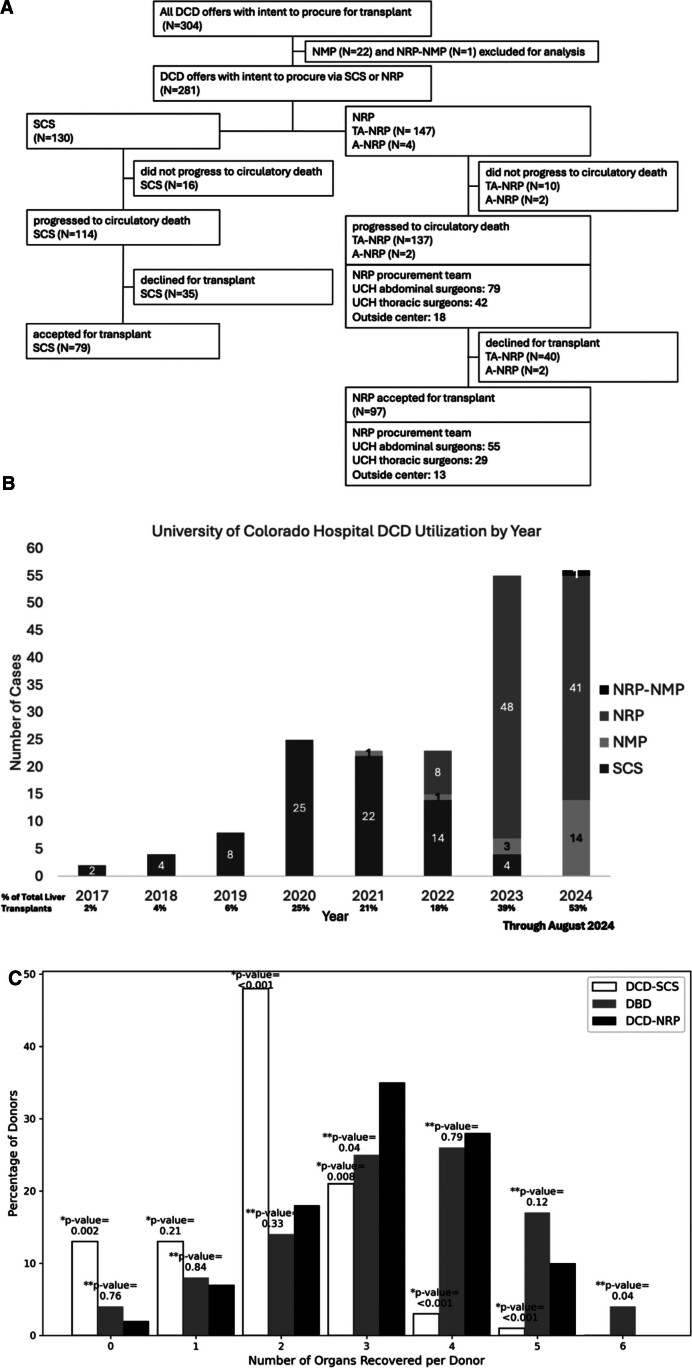
Institutional DCD liver transplant offers and transplant volume: SCS vs NRP and organ procurement organization organ yield per donor type. A, Flow diagram of DCD offers that UCH attended with the intent to procure the liver for transplant. B, The yearly volume of DCD LT has expanded; we performed 97 DCD-NRP and 79 DCD-SCS LT from January 2017 to August 2024. UCH’s first DCD-NRP liver accepted for transplant was in February 2022, with a procurement performed by an outside center. The first DCD TA-NRP liver procurement performed by the UCH team was in October 2022. C, Number of organs recovered stratified by procurement technique for the local organ procurement organization during the study period. A Fisher exact test was used to compare organ yield by donor type. Comparisons by donor type are indicated as *DCD-SCS vs DCD-NRP and **DCD-NRP vs DBD. A-NRP, abdominal normothermic regional perfusion; DBD, donation after brain death; DCD, donation after circulatory death; LT, liver transplant; NMP, normothermic machine perfusion; SCS, static cold storage; TA-NRP, thoracoabdominal NRP; UCH, University of Colorado Hospital.

There were 304 potential DCD donors to which UCH sent a team to procure a liver for transplant (Figure 1A). Excluding 22 NMP cases, the utilization rate of livers from DCDs who progressed when NRP was and was not available was 71% and 69%, respectively. All accepted SCS livers were procured by UCH surgeons. All accepted NRP livers were via the thoracoabdominal approach. Of the DCD TA-NRP livers accepted for transplant, UCH abdominal surgeons performed 55 cannulations (56.7%). No NRP livers were declined because of technical failures. The proportion of total LT volume attributed to DCD at our institution increased from 2% in 2017 to 53% in August 2024 (Figure 1B).

### OPO Organ Yield per Donor Type

The organ yield for the local OPO for DCD via SCS was statistically significantly lower than that of DCD via NRP (1.91 versus 3.36, *P* < 0.001) during the study period. The organ yield for DCD via NRP was not statistically different compared with that of DBD (3.31 versus 3.36, *P* = 0.08; Table 1). The percentage of donors yielding 3 organs was higher for DCD via NRP compared with DCD via SCS (35% versus 21%, *P* = 0.008) and compared with DBD (35% versus 25%, *P* = 0.04; Figure 1C).

**TABLE 1.** Organ procurement organization organ yield across procurement method, January 2017–July 2024

	DCD-SCS	DCD-NRP	*P*	DBD	DCD-NRP	*P*
Kidney	901 (78%)	164 (82%)	0.51	822 (82%)	164 (82%)	0.99
Liver	142 (25%)	97 (82%)	<0.001	790 (79%)	97 (82%)	0.58
Heart	41 (7%)	50 (47%)	<0.001	511 (50%)	50 (47%)	0.57
Lung	17 (3%)	15 (13%)	<0.001	230 (26%)	15 (13%)	0.01
Organ yield	1.91	3.36	<0.001	3.31	3.36	0.08

Organ yield and percentage of organs recovered by procurement technique for the local organ procurement organization. The mean number of organs recovered from DBD was 3.31 relative to a mean of 3.07 for NRP (*P* = 0.08) and 1.91 for DCD-SCS (*P* < 0.001 with NRP as reference). Due to low utilization, the pancreas and intestine were excluded from the organ yield analyses.

DBD, donation after brain death; DCD, donation after circulatory death; NRP, normothermic regional perfusion; SCS, static cold storage.

### DCD Donor and Procurement Characteristics of Accepted Livers for Transplant: SCS vs NRP

NRP donors were older (41 [30–56] versus 33 [24–40] y, *P* < 0.001), had a higher body mass index (28.6 [24.7–31.7] versus 24.8 [22.1–29.2] kg/m^2^, *P* = 0.002), and had higher DRI (2.44 [2.02–2.82] versus 2.17 [1.97–2.30], *P* = 0.002) compared with SCS donors. Transplanted NRP livers were from donors with longer total minutes of WIT (25 [20–30] versus 20 [17–23], *P* < 0.001) and longer fWIT based on OPTN, ASTS, and International Liver Transplant Society guidelines (Table 2). The longest fWIT for an accepted NRP liver was 49 min by OPTN criteria (**Figure S3, SDC,** http://links.lww.com/TXD/A740). NRP recipients experienced shorter cold ischemia time (CIT; 243 min [214–288] versus 289 min [253–339], *P* < 0.001; Table 2). The mean UK DCD Risk Score was higher for NRP compared with SCS donor-recipient pairs (4.2 ± 2.9 versus 3.2 ± 2.3, *P* = 0.008). Twenty-three NRP donor-recipient pairs (23.7%) were considered high risk or futile (UK DCD Risk Score >5) and 5 of these NRP livers were used for retransplants.

**TABLE 2.** Donor and procurement characteristics of accepted livers for transplant in DCD: NRP vs SCS

	NRP (N = 97)	SCS (N = 79)	*P*
Donor characteristics			
Age, y	41 (30–56)	33 (24–40)	0.0001
Sex, male	69 (71.13%)	56 (70.89%)	1
BMI, kg/m^2^	28.6 (24.7–31.7)	24.8 (22.1–29.2)	0.002
Demographics			0.89^*a*^
White	72 (74.23%)	59 (75%)	
Hispanic/Latino	16 (16.49%)	12 (15%)	
Black or African American	6 (6.19%)	6 (8)	
Asian/Hawaiian Pacific Islander	2 (2.06%)	2 (2)	
American Indian/Native American	1 (1.03%)	0 (0%)	
Medical and social history			
Heavy EtOH	25 (25.77%)	19 (24%)	0.93
HCV NAT+	1 (1.03%)	0 (0%)	1
Cause of death			0.13
Anoxia	35 (36.08%)	39 (49%)	
Cerebrovascular accident	23 (23.71%	14 (18%)	
Head trauma	34 (35.05%)	24 (30%)	
Other	5 (5.15%)	2 (3%)	
Donor hospital DCI^37^			0.57^*a*^
Distressed	2 (2.06%)	8 (10%)	
At risk	41 (42.27%)	36 (46%)	
Mid-tier	17 (17.52%)	9 (11%)	
Comfortable	12 (12.37%)	10 (13%)	
Prosperous	25 (25.77%)	16 (20%)	
Predonation hepatic function panel			
Terminal AST, U/L	49 (31–78)	51 (32–87)	0.34
Terminal ALT, U/L	36 (21–74)	45 (25–98)	0.10
Terminal TB, U/L	0.5 (0.4–0.8)	0.5 (0.4–0.8)	0.36
Procurement characteristics			
Total WIT, min	25 (20–30)	20 (17–23)	<0.001
fWIT, min			
ASTS (SBP <50 mm Hg or O_2_ sat <70%)	20 (16–24)	16 (14–19)	<0.001
ILTS (MAP <60 mm Hg or O_2_ sat <80%)	21 (17–26)	17 (15–20)	<0.001
OPTN (SBP <80 mm Hg or O_2_ sat <80%)	21 (17–26)	17 (14–19)	<0.001
Cold ischemia time, min	243 (214–288)	289 (253–339)	<0.001
DRI^34^	2.44 (2.02–2.82)	2.17 (1.97–2.30)	0.002
Simultaneous operation			
Kidney transplant	8 (8.24%)	3 (3.80%)	0.36
Pancreaticoduodenectomy	1 (1.03%)	0 (0%)	1

Categorical values are presented as n (%); continuous values are presented as median (interquartile range).

^*a*^Chi-square *P* value.

ALT, alanine transaminase; AST, aspartate transferase; ASTS, American Society of Transplant Surgery; BMI, body mass index; DCI, Distressed Community Index; DRI, Donor Risk Index; EtOH, alcohol use; fWIT, functional warm ischemia time; HCV, hepatitis C virus; ILTS, International Liver Transplant Society; MAP, mean arterial pressure; NAT, nucleic acid test; NRP, normothermic regional perfusion; O_2_ sat, oxygen saturation; OPTN, Organ Procurement and Transplant Network; SBP, systolic blood pressure; SCS, static cold storage; TB, total bilirubin.

### NRP Donor and Procurement Characteristics: Accepted vs Declined Livers

Accepted NRP livers had fewer minutes of total WIT (25 [20–30] versus 31 [24–47], *P* = 0.006) and fewer minutes of fWIT following ASTS, International Liver Transplant Society, and OPTN criteria. Trends in WIT were variable among NRP and SCS cases (**Figure S3, SDC** http://links.lww.com/TXD/A740). The median NRP cannulation time between accepted and declined livers were 6 (4–7) min versus 7 (4–9) min, respectively. Accepted NRP allografts had more minutes of perfusion (104 [90–119] versus 85 [94–105], *P* = 0.004). The median amount of blood, bicarbonate, normal saline, and lactated ringer administered was 250 (250–500) mL, 25 (12.5–37.5) mEq, 0 (0–0) mL, and 0 (0–0) mL in accepted NRP allografts, respectively. There were no significant differences between accepted and declined NRP livers regarding transfusions, administration of medications or fluids, or pump flows during NRP (Table 3). There was no statistically significant association between the amount of blood transfused and lactate concentrations during NRP. Moreover, there were no statistically significant associations between the amount of medications or fluid administered and lactate concentrations.

**TABLE 3.** Donor and normothermic regional perfusion procurement characteristics in donation after circulatory death: accepted vs declined livers

	Accepted (N = 97)	Declined (N = 40)	*P*
Donor characteristics			
Age, y	41 (30–56)	46 (37–58)	0.73
Sex, male	69 (71.13%)	25 (62.5%)	0.43
BMI, kg/m^2^	28.7 (24.7–31.7)	27.7 (23.6–31.8)	0.76
Medical and social history			
Heavy EtOH	25 (25.77%)	11 (27%)	0.79
HCV NAT+	1 (1.03%)	2 (5%)	0.14
Cause of death			0.22^*a*^
Anoxia	35 (36.08%)	19 (47.5%)	
Cerebrovascular accident	23 (23.71%)	12 (30%)	
Head trauma	34 (35.05%)	7 (17.5%)	
Other	5 (5.15%)	2 (5%)	
Procurement characteristics			
Total WIT, min	25 (20–30)	31 (24–47)	0.006
fWIT, min			
ASTS (SBP <50 mm Hg or O_2_ sat<70%)	20 (16–24)	28 (19–45)	0.002
ILTS (MAP <60 mm Hg or O_2_ sat<80%)	21 (17–26)	28 (21–46)	0.002
OPTN (SBP <80 mm Hg or O_2_ sat <80%)	21 (17–26)	28 (20–46)	0.002
Perfusion parameters			
Time to cannulate NRP, min	6 (4–7)	7 (4–9)	0.77
Perfusion time, min	104 (90–119)	85 (94–105)	0.004
pRBC administered, mL	250 (250–500)	250 (250–500)	0.39
Crystalloid, mL			
Normal saline, mL	0 (0–0)	0 (0–0)	0.93
Lactated Ringer, mL	0 (0–0)	0 (0–0)	0.89
Bicarbonate administered, mEq	25 (12.5–37.5)	50 (0–50)	0.18
Calcium chloride, mL	1 (0.75–1)	1 (0–1)	0.68
Phenylephrine, µg	150 (100–200)	200 (100–250)	0.74
Vasopressin, units	1 (1–2)	2 (1.25–2.0)	0.40
Arterial flow, L/min	3.79 (3.32–4.40)	3.90 (3.39–4.27)	0.79
Terminal serum pH, n	78	26	
Terminal serum pH	7.36 (7.31–7.41)	7.30 (7.23–7.37)	0.015
Terminal serum bicarbonate, n	66	17	
Terminal serum bicarbonate, mEq/L	21.4 (19.0–24.0)	19.4 (16.7–20.6)	0.07
Terminal serum glucose, n	67	24	
Terminal serum glucose, mg/dL	198 (167–234)	237 (157–270)	0.48
Hepatocellular parameters			
Lactate, n	77	25	
Peak lactate, mg/dL	8.8 (7.6–9.8)	10.1 (8.8–11)	0.004
Delta lactate, mg/dL	–3.2 (–4.0 to –1.92)	–2.2 (–4.05 to –0.66)	0.04
AST, n	75	22	
Peak AST, U/L	132 (86–255)	208 (81–440)	0.015
Delta AST, U/L	58 (28–126)	225 (67–450)	0.08
ALT, n	75	21	
Peak ALT, U/L	85 (55–126)	90 (55–453)	0.65
Delta ALT, U/L	31 (14–76)	125 (15–426)	0.31
Cholangiocellular parameters			
Terminal biliary pH, n	58	13	
Terminal biliary pH	7.7 (7.6–7.7)	7.64 (7.41–7.70)	0.39
Biliary bicarbonate, n	15	5	
Terminal biliary bicarbonate, mEq/L	22.5 (20.7–27.2)	10.2 (7.41–11.6)	0.004
Peak biliary bicarbonate, mEq/L	23 (21.7–29.9)	13.3 (7.7–16.7)	0.01
Terminal biliary glucose, n	37	10	
Terminal biliary glucose, mg/dL	20 (20–20)	20 (20–20)	0.87
Nadir biliary glucose, mg/dL	20 (20–20)	20 (20–20)	1
Macrosteatosis on procurement biopsy, n	60	16	
0%	26 (44%)	6 (37.5%)	0.78
1%–10%	23 (38%)	3 (18.75%)	0.23
11%–20%	6 (10%)	1 (6.25%)	1
21%–30%	1 (2%)	4 (25%)	0.006
31%–40%	2 (3%)	0 (0%)	1
>40%	2 (3%)	2 (12.5%)	0.19

Categorical values are presented as n (%); continuous values are presented as median (interquartile range). Delta calculated via terminal minus initial value.

^*a*^Chi-square *P* value.

ALT, alanine transaminase; AST, aspartate transferase; ASTS, American Society of Transplant Surgery; BMI, body mass index; EtOH, alcohol use; fWIT, functional warm ischemia time; HCV, hepatitis C virus; ILTS, International Liver Transplant Society; MAP, mean arterial pressure; NAT, nucleic acid test; NRP, normothermic regional perfusion; O_2_ sat, oxygen saturation; OPTN, Organ Procurement and Transplant Network; pRBC, packed red blood cell; SBP, systolic blood pressure.

Accepted NRP livers had lower peak lactate (8.8 [7.9–9.8] versus 10.1 [8.8–11.0] mg/dL, *P* = 0.004), a larger delta in terminal minus initial lactate (–3.4 [–4.0 to –1.92] versus –2.2 [–4.05 to –0.66] mg/dL, *P* = 0.04), a lower peak aspartate transferase (32 [86–255] versus 208 [81–440] U/L, *P* = 0.015), a higher terminal biliary bicarbonate (22.5 [20.7–27.2] versus 10.2 [7.41–11.6] mEq/L, *P* = 0.004), and higher peak biliary bicarbonate (23 [21.7–29.9] versus 13.3 [7.7–16.7] mEq/L, *P* = 0.01; Table 3; **Figure S4, SDC,** http://links.lww.com/TXD/A740).

### Recipient Characteristics and Perioperative Resource Utilization of DCD LT: SCS vs NRP

There were fewer hours of recipient surgery time for NRP compared with SCS allografts (5.37 [4.53–6.63] versus 6 [5.23–7.57], *P* = 0.015). Intraoperatively, NRP recipients required less milliliters of fresh frozen plasma (FFP; 2750 [1500–5000] versus 4500 [2500–7000], *P* = 0.003), platelets (PLTs; 200 [0–400] versus 400 [400–600], *P* < 0.001), and cryoprecipitate (0 [0–100] versus 100 [3–200], *P* < 0.001) compared with SCS recipients. Postoperatively, NRP recipients required less milliliters of packed red blood cells (700 [0–1400] versus 1400 [350–2450], *P* = 0.001), PLTs (0 [0–300] versus 200 [0–600], *P* = 0.007), and cryoprecipitate (0 [0–0] versus 0 [0–100], *P* = 0.008). A majority of both DCD groups were extubated in the operating room, recovered in the postanesthesia care unit, and transferred to the floor (Table 4).

**TABLE 4.** Recipient characteristics and perioperative resource utilization of donation after circulatory death liver transplant: NRP vs SCS

	NRP (N = 97)	SCS (N = 79)	*P*
Recipient characteristics			
Age, y	54 (48–60)	56 (46–62)	0.44
Sex, male, n (%)	63 (64.9)	52 (65.8)	1^*a*^
BMI, kg/m^2^	26.9 (24.0–31.0)	27.4 (25.1–31.1)	0.64
Demographics, n (%)			0.17^*a*^
White	56 (57.73)	52 (65.82)	
Hispanic/Latino	31 (31.96)	18 (22.78)	
Black or African American	2 (2.06)	3 (3.80)	
Asian/Hawaiian Pacific Islander	1 (1.03)	4 (5.06)	
American Indian/Native American	1 (1.03)	0 (0)	
Other	0	0 ()	
Primary indication for LT, n (%)			
EtOH	37 (38.14)	28 (35.44)	0.83
NASH	16 (16.49)	12 (15.19)	0.98
HCC	17 (17.52)	15 (18.99)	0.72
Biliary disease	12 (12.37)	6 (7.59)	0.43
Other	15 (15.46)	18 (22.78)	0.26
Medical/surgical history, n (%)			
Previous abdominal surgery	47 (48.45)	41 (51.90)	0.79
Portal vein thrombosis	14 (14.43)	19 (24.05)	0.15
History of prior LT	5 (5.15)	0 (0)	0.11
Pretransplant location, n (%)			
Home	86 (88.66)	74 (93.67)	0.37
Inpatient floor	9 (9.28)	4 (5.06)	0.44
Intensive care unit	2 (2.06)	1 (1.27)	1
Need for pretransplant RRT, n (%)	12 (12.37)	2 (2.53)	0.03
UNOS allocation MELD-Na score	22 (18–26)	22 (17–25)	0.13
Laboratory MELD-Na score	20 (16–23)	20 (17–24)	0.75
Operative characteristics			
Anastomotic WIT, min	28 (25–32)	29 (27–33)	0.15
Recipient surgery time, h	5.37 (4.53–6.63)	6 (5.23–7.57)	0.015
Intraoperative transfusion, mL			
pRBC	2450 (1080–4900)	2800 (1775–5500)	0.27
FFP	2750 (1500–5000)	4500 (2500–7000)	0.003
Platelets	200 (0–400)	400 (400–600)	<0.001
Cryoprecipitate	0 (0–100)	100 (3–200)	<0.001
Postoperative disposition, n (%)			
PACU then inpatient floor	63 (64.9)	47 (59.49)	0.75
SICU	34 (35.1)	32 (40.51)	0.75
Postoperative transfusion, mL			
pRBC	700 (0–1400)	1400 (350–2450)	0.001
FFP	0 (0–500)	250 (0–500)	0.14
Platelets	0 (0–300)	200 (0–600)	0.007
Cryoprecipitate	0 (0–0)	0 (0–100)	0.008
Need for posttransplant RRT	19 (19.59)	11 (13.92)	0.43

Categorical values are presented as n (%); continuous values are presented as median (interquartile range).

^*a*^Chi-square *P* value.

BMI, body mass index; EtOH, alcohol use; FFP, fresh frozen plasma; HCC, hepatocellular carcinoma; LT, liver transplant; MELD-Na, model for end-stage liver disease-sodium; NASH, nonalcoholic steatohepatitis; NRP, normothermic regional perfusion; PACU, postanesthesia care unit; pRBC, packed red blood cell; RRT, renal replacement therapy; SCS, static cold storage; SICU, surgical intensive care unit; UNOS, United Network for Organ Sharing; WIT, warm ischemia time.

### Postoperative Outcomes of DCD LT: SCS vs NRP

**FIGURE 2.**
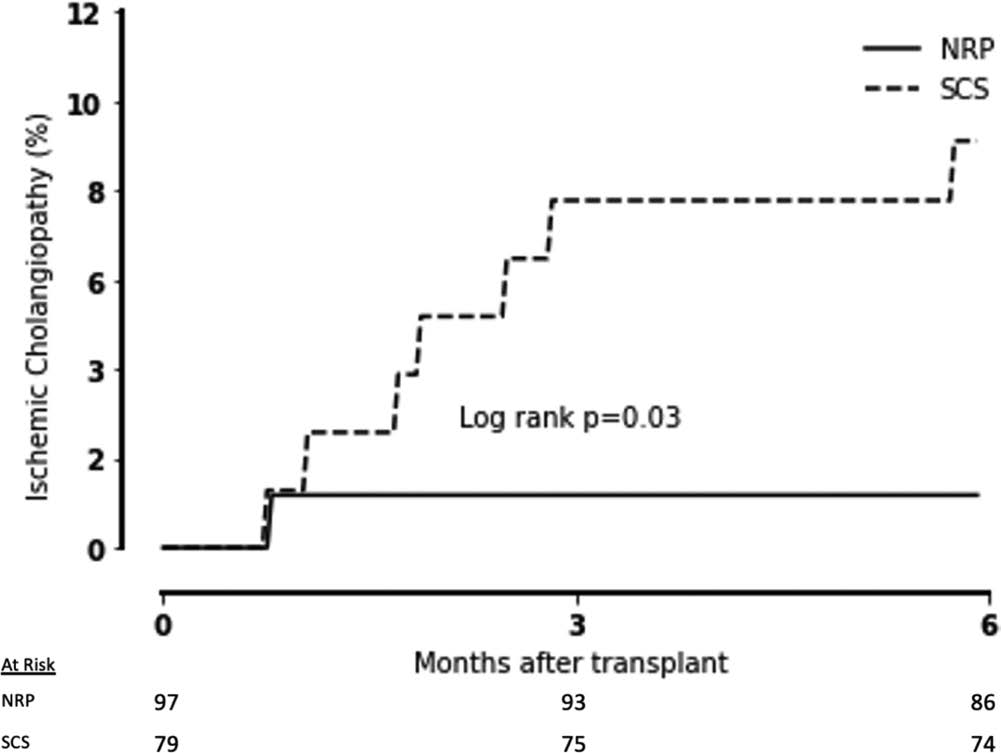
Cumulative incidence of ischemic cholangiopathy at 6 mo. Cumulative incidence of ischemic cholangiopathy of liver transplant recipients with at least 6-mo of follow-up stratified by NRP (n = 86) and SCS (n = 74). There was 1 case of ischemic cholangiopathy in the NRP group at day 24. NRP, normothermic regional perfusion; SCS, static cold storage.

The median follow-up time was 382 (254–599) d and 1374 (1067–1708) d for SCS and NRP groups, respectively. There were 86 NRP recipients and 74 SCS recipients with at least 6 mo of follow-up. The 6-mo rate of IC was lower for NRP compared with SCS (1.2% versus 9.5%, *P* = 0.03). IC classification for SCS included 2 diffuse necrosis, 4 multifocal progressive, and 1 minor form. There was 1 episode of IC for NRP and this was diffuse necrosis (Table 5). There was a significant difference in the cumulative incidence of IC (*P* = 0.03; Figure 2).

**TABLE 5.** Postoperative outcomes of donation after circulatory death: NRP vs SCS

	NRP (N = 97)	SCS (N = 79)	*P*
EAD,^5^ n (%)	44 (45.36)	39 (49.36)	0.70^*a*^
L-GrAFT^40^	–3.3 (–3.9 to –2.9)	–3.1 (–3.5 to –2.4)	0.0002
MEAF^41^	4.7 (3.9–5.9)	5.2 (3.9–6.3)	0.26
Peak ALT	666 (433–1363)	652 (372–926)	0.25
Peak AST	1330 (790–2720)	1793 (1000–2892)	0.26
Peak TB	5.7 (3.9–8.2)	5.9 (3.9–9.9)	0.26
Peak INR	1.8 (1.5–2.1)	1.8 (1.6–2.1)	0.48
TB POD 7	1.9 (1.2–3.2)	2.2 (1.6–5.7)	0.004
INR POD 7	1.1 (1.1–1.3)	1.2 (1.1–1.3)	0.02
Hospital length of stay, d	9 (6–12.25)	9 (7–13)	0.91
ICU length of stay, d	0 (0–2)	1 (0–4)	0.18
Return to OR reason, n (%)			
Staged OLT	5 (5.15)	7 (8.86)	0.50
Unplanned			
Bleeding	13 (13.40)	16 (20.25)	0.31
Bowel perforation	1 (1.03)	0 (0)	1
HAT, n (%)	0 (0)	3 (3.80)	0.18
PNF, n (%)	2 (2.06)	0 (0)	0.57
Retransplanted	3 (3)	3 (3.80)	0.88
Clinically significant biliary complications, n (%)			
Median follow-up days post liver transplant	382 (254–599)	1374 (1067–1708)	<0.001
Anastomotic leak	4 (4.12)	2 (3)	0.87
Anastomotic stricture	1 (1.03)	5 (6)	0.13
At risk of IC at 6 mo, n (%)	86 (88.6)	74 (93.6)	
Median day of nonanastomotic stricture (IC) diagnosis	24^*b*^	57 (32–86)	
Nonanastomotic stricture (IC) at 6 mo	1 (1.2)	7 (9.5)	0.03
Nonanastomotic stricture (IC) Classification^44^			
Diffuse necrosis	1	2	
Multifocal progressive		4	
Confluence dominant			
Minor form		1	
Readmissions within 90 d, n (%)	45 (46.39)	44 (55.7)	0.28
Clavien-Dindo grade ≥IIIb,^38^ n (%)	7 (7.22)	16 (20.25)	0.02

Categorical values are presented as n (%); continuous values are presented as median (interquartile range).

^*a*^Chi-square *P* value.

^*b*^One case of ischemic cholangiopathy diagnosed on POD 24.

ALT, alanine transaminase; AST, aspartate transferase; EAD, early allograft dysfunction; HAT, hepatic artery thrombosis; IC, ischemic cholangiopathy; ICU, intensive care unit; INR, international normalized ratio; L-GrAFT, liver graft assessment following transplantation; MEAF, Model for Early Allograft Function Scoring; NRP, normothermic regional perfusion; OLT, orthotopic liver transplant; OR, operating room; POD, postoperative day; PNF, primary nonfunction; SCS, static cold storage; TB, total bilirubin.

NRP allografts had a lower risk of graft loss according to the L-GrAFT score (–3.3 [–3.9 to –2.9) versus –3.1 [–3.5 to –2.4], *P* = 0.0002). Following an L-GrAFT nomogram, this corresponds to a 15% decrease in risk of 3-mo graft failure for NRP compared with SCS.^45^ There was no difference in EAD or MEAF between NRP and SCS livers. Postoperative day (POD) 7 total bilirubin was less in NRP recipients (1.9 [1.2–3.2] versus 2.2 [1.6–5.7], *P* = 0.004) as was POD 7 international normalized ratio (1.1 [1.1–1.3] versus 1.2 [1.1–1.3], *P* = 0.02. There were no differences in hepatic artery thrombosis, PNF, return to the operating room, or hospital readmission between groups. Readmission for Clavien-Dindo grade IIIb complications was higher for SCS compared with NRP livers (16% versus 7%, *P* = 0.02; Table 5).

There were no differences in allograft or patient survival at 12-mo of follow-up (noncensored for death 93% versus 91%, *P* = 0.89; censored for death 96% versus 95%, *P* = 0.75; patient survival 97% versus 95%, *P* = 0.43; **Figure S1, SDC,** http://links.lww.com/TXD/A740). The NRP cohort had 5 all-cause graft losses and 2 all-cause recipient mortalities. One mortality was on POD 100 in the setting of multiorgan sepsis unrelated to NRP. The other mortality was on POD 132 secondary to an aspiration event unrelated to NRP and the recipient passed with excellent graft function. There were 2 PNFs (2%) despite reassuring biochemical testing during NRP. Procurement notes reveal one allograft felt somewhat firm and the other did not make bile until the end of the NRP run. Both patients underwent retransplant, one with a DBD and the other with TA-NRP DCD and are doing well. There was one case of diffuse necrosis IC in the NRP cohort, and this resulted in graft loss and subsequent retransplant with a DBD. This NRP allograft was procured by an outside hospital with acceptable macroscopic characteristics; however, biochemical testing was not recorded for review (**Table S1, SDC,** http://links.lww.com/TXD/A740).

## DISCUSSION

Our real-world experience illustrates NRP for DCD LT to be a disruptive innovation because the implementation of an NRP program facilitated an increase in utilization. Despite transplanting higher-risk livers and donor-recipient pairs with NRP compared with our SCS cohort according to the UK DCD Risk Score, we demonstrated a decrease in posttransplant IC rate at 6 mo and maintained excellent allograft and patient survival at 12 mo. Recipients of NRP livers had a 15% risk reduction in graft loss compared with SCS livers following the L-GrAFT score. We extend international reports of the benefits of NRP via providing granular procurement and recipient outcomes for the largest known series of TA-NRP for LT. Moreover, TA-NRP has increased organ yield per donor compared with SCS, which is similar to that of DBD.

Often considered marginal grafts, DCD livers have been discarded at high rates in the United States, with an estimated utilization rate of 27.1% in the era of SCS.^46-48^ With the advent of NRP, DCD allografts now account for >55% of our total LT volume. NRP allows assessing allografts in situ before declining solely based on reflex of preprocurement variables. For example, our center practice was to routinely decline allografts from SCS donors based on a variable thought to be high risk, such as WIT of >30 min. Indeed, in 1 NRP case, we transplanted a liver with fWIT of 49 min with an excellent outcome. Similar to the Spanish, our practice has evolved to consider an NRP liver for any listed recipient and a well-selected SCS liver for high-risk recipients.^19,49^ For example, we used NRP livers for a higher proportion of candidates with preoperative renal replacement therapy compared with SCS (12.3% versus 2.5%, *P* = 0.03).

Our statistically significantly lower 6-mo IC rate for NRP compared with SCS (1.4% versus 8.86%, *P* = 0.03) is consistent with others demonstrating the benefit of NRP on biliary outcomes.^19,20,22,26^ The single case of IC was diagnosed on POD 24 in the setting of acute liver failure requiring retransplant. Internationally, IC rates after DCD-NRP are variable (0%–11%) and likely reflect differing procurement practices, sample sizes, and confounders of donor-recipient matching; nevertheless, our results are on par with the contemporary literature. A Dutch center described an IC rate of 11% in 20 A-NRP cases; a UK group reported a biliary stricture rate of 6% in 69 cases; and the Spanish documented a rate of 1% for ischemic-type biliary lesions in 545 A-NRP cases. A recent multicenter US experience documented an IC rate of 0% in 104 cases; however, graft losses and death occurring <90 d from transplant were excluded.^15,19,22,26^

This study supports expanding criteria for NRP utilization. Compared with our SCS cohort, NRP facilitated our use of donors with increased DRI, increased fWIT, and transplant of higher-risk donor-recipient pairs by UK DCD Risk Score while maintaining acceptable outcomes. Our NRP recipients were sicker compared with others as our median laboratory MELD of 20 is higher than that of 12 reported in the large Spanish series.^19^ Additionally, our NRP cohort had only 16% HCC compared with larger proportions of 26%–55%.^15,19,22,26^ The UCH NRP practice pattern is likely more progressive compared with earlier US experience as we did not decline livers solely based on macrosteatosis and our median NRP donor age of 41 y is a decade older than 25 y and 30.5 y reported in prior US multicenter NRP studies.^15,29^ We acknowledge that our median NRP donor age is less than that of contemporary European experiences of DCD-NRP.^19,22,26^ We are eager to learn from the international community regarding how best to expand the program and potentially use sequential perfusion technologies to facilitate the transplant of allografts from donors older than 70 y.^28^

During a 20-mo period, our group transplanted 54 abdominal-only DCD offers via TA-NRP compared with the 27 A-NRP cases amassed by 7 centers in the multi-institutional US effort. It is likely that a large percentage of previously reported TA-NRP LT in the United States were from donors in which thoracic organs were also allocated and thus from overall lower-risk donors.^15^ The difference in entertaining abdominal-only offers may also be explained by the evolution of NRP practice since an early landmark US publication.^15^

In a testament to our perioperative teams, 65% of NRP and 59% of SCS DCD LTs were extubated in the operating room, recovered in the postanesthesia care unit, and transferred to the floor. Observed improvements in perioperative outcomes could be attributed to better graft selection during the NRP era; however, differences in DRI and UK DCD scores suggest that NRP allografts and donor-recipient combinations on average were higher risk. We suspect that NRP facilitated improved perioperative outcomes because of the impact of dynamic preservation during NRP.^50^ NRP compared with SCS recipients received less intraoperative fresh frozen plasma, PLTs, and cryoprecipitate, suggesting NRP may mitigate coagulopathy. Moreover, NRP recipients received less transfusion of postoperative packed red blood cells, PLTs, and cryoprecipitate compared with SCS, which likely reflects sustained allograft function. Although we did not observe differences in EAD of MEAF rates between groups, the NRP group had 15% less risk of graft failure compared with SCS according to the L-GrAFT score. We agree that further research is warranted to validate the accuracy of risk scores in predicting graft loss in DCD via NRP.^51^

TA-NRP was favored by our center as it allows for increased organ yield per DCD donor for both thoracic and abdominal organs.^16^ An advantage of the TA approach is that exposure for cannulation in the chest is technically more feasible than in the abdomen for donors with increased body mass index or past abdominal surgical history. Nevertheless, A-NRP was an option if the potential DCD had previous cardiothoracic surgery, which made TA-NRP cannulation nonfeasible.

We followed perfusion, hepatocyte, and cholangiocyte parameters during NRP to assess allograft suitability.^21,26,52^ These 3 biochemical assessments were adjuncts to clinical judgment and have not been previously reported as a group in the North American NRP literature. In addition, we report information regarding flow, transfusion of blood, and administration of medications such as bicarbonate during NRP. We did not find any significant associations between these NRP adjuncts and biochemical assessments during NRP. However, there were notable differences in viability assessments. Namely, transplanted livers had lower peak lactate, a larger delta lactate, a lower peak aspartate transferase, a higher terminal and peak biliary bicarbonate during NRP. The influence of each parameter on utilization is unclear and warrants further investigation, but these data are useful in benchmarking.

The community’s experience with NMP LT allows us to hypothesize that lactate clearance serves as a proxy of liver function during NRP.^53,54^ However, we were not able to describe a clinically meaningful lactate trend.^26,32^ The data illustrate decreasing lactate for both accepted and declined livers. Indeed, 2 recipients with PNF demonstrated decreasing lactate. We rarely saw lactate decrease with a sharp slope, potentially because lactate-concentrated blood returned to the NRP circuit from hypoperfused tissue.^26^ We did not appreciate an association between blood transfusion or administration of bicarbonate and lactate trends on NRP. Lactate assessment in NMP has been useful because NMP is a closed ex situ system, yet others have reported PNF in NMP despite decreasing lactate.^55^ Lactate clearance occurs in zone 1 of periportal hepatocytes, which is the last zone to experience a depletion of oxygen stores; thus, lactate may not increase until any injury to all hepatocyte zones.^56^ We caution that following lactate alone for in situ viability assessment in NRP is not sufficient.

We were hesitant to accept livers with increasing transaminases on NRP, suspecting these laboratory values reflected ischemia/reperfusion injury, but we recognize that elevation in these laboratory values may be confounded by secretion from other tissues in the body. As we perform NRP in donors who progressed to circulatory death, some transaminase elevation may be secondary to the process of circulatory arrest.^57^

There is debate in the LT perfusion community regarding the inclusion of bile during viability assessment.^58-60^ Nevertheless, our study illustrates clinically meaningful differences between accepted versus declined NRP livers regarding terminal and peak bile bicarbonate. We were able to sample bile at different time points during NRP to ensure that assessment was not only based on bile made before initiation of NRP. The terminal bile sample was collected just before the organ acceptance decision; as our median perfusion time for accepted livers was 104 min, it is unlikely that the bile collected at the end of NRP was the same bile made before the declaration of death. Accepted livers had median terminal and peak bile bicarbonate values that were 10 mEq/L greater compared with declined livers. Well-functioning cholangiocytes secrete bicarbonate to mitigate the detrimental effects of bile salts and subsequent cholangiopathy from developing what is coined as a “bicarbonate umbrella.”^61^ Bile production seems to be especially important in that both PNF cases had poor bile production despite reassuring lactate and liver function test trends during NRP. Indeed, other NRP experiences have commented that bile production was associated with acceptance.^26^ As such, biliary evaluation may serve as a proxy for other clinical assessments specific to the liver.

Literature on bile assessment of livers on NMP suggest measuring parameters at time points of >2 h, including biliary lactate dehydrogenase, and considering ratios of biliary values compared with those in the perfusate.^55,59^ It is unclear whether viability criteria from NMP, an ex situ technology, are generalizable to NRP, an in situ technology. Our NRP time was limited to ≤120 min, given logistical constraints and so additional measurements were not possible. Nevertheless, analyses of the biliary proteome during NMP support the promise of incorporating biliary biomarkers in the viability assessment of NRP livers.^62^

NRP liver allograft acceptance criteria are variable at the international level.^33^ If we are going to identify the limits of NRP, then it is prudent to identify markers of viability assessment that are sensitive and specific to the allograft and can be measured in real time. Assessment of mitochondrial injury of the allografts while on NRP via flavin mononucleotide is a growing area of interest.^63^ We are eager to collaborate to improve the current data collection system in the United States, as the Scientific Registry of Transplant Recipients does not capture NRP as a procurement method, and DonorNet does not record NRP variables. The Colorado Organ Assessment Form may serve as a framework for data collection in the United States similar to that which is implemented in the United Kingdom.^64^ As TA-NRP was able to increase organ yield for liver and other organs, additional processes should be developed to safeguard the quality of all organs to be transplanted.

There continues to be ethical discussion regarding NRP for DCD organ recovery, with debate focused on nonmaleficence.^65^ Our center practice conforms to the published technical and ethical standards of the ASTS.^9-12^ We agree with our colleagues to honor donor autonomy and that NRP is indeed a postmortem perfusion technique of potential allografts for transplant and does not violate the Dead Donor Rule.^12^ Moreover, our series confirms the benefits of NRP in the context of LT as there is an increased likelihood of using gifts of life from a DCD donor and improved outcomes in a recipient. As such, we contend there may be unintended harm in not optimizing the potential to transplant allografts and unnecessary risk to a potential DCD LT recipient if NRP were not considered.

This study is limited by its retrospective nature at a single center and by the low event rate of the primary outcome, IC, to conduct matched, risk-adjusted analyses. The follow-up period for IC in our study was 6 mo, which may have missed potential occult cases. Our center did not have a protocol for routine surveillance of IC. However, we engaged in diligent laboratory and clinical follow-up for all recipients; 88.6% of the NRP and 93.6% of the SCS cohorts reached a 6-mo follow-up. A multicenter assessment of IC in the United States demonstrated the overall median (interquartile range) number of days from DCD LT to the diagnosis of IC was 36.5 d (21–65).^44^ Specifically, the authors reported the spectrum of IC classifications was diagnosed in <6 mo as median days from DCD LT for diffuse necrosis and minor form were 21 (9–56) d and 73 (30–169) d, respectively.^44^ Our series captured IC classified by diffuse necrosis, multifocal progressive, and minor form. As such, we surmise that our endpoint of 6 mo would be sensitive to the diagnosis of a clinically significant episode of IC.

The nonsignificant differences in secondary outcomes between groups are at risk of type 2 error. Our utilization rates suggest the ongoing benefits of perfusion technology; however, they refer to the number of accepted DCD offers in which the DCD process was initiated and do account for all potential offers. Nevertheless, it is likely that we will be able to sustain a high volume of DCD LT utilization because of our commitment to NRP. This parallels the international literature as countries that use perfusion technology regularly have increased DCD utilization.^48^ Consistent with the literature, it is difficult to recommend 1 set of evaluation criteria during NRP based on these data, and perhaps our graft selections were biased by other reports.^33^ The lower incidence of IC via DCD-NRP might be secondary to a median difference in CIT of 46 min between groups. However, the median CIT for both DCD via SCS and NRP is less than a threshold of 6 h, which is a risk factor for poor posttransplant outcomes.^35^

We report a large DCD LT TA-NRP experience for the United States and provide granular data regarding allograft viability testing and outcomes to serve as a benchmark for future studies. In our center experience, NRP has facilitated increased use of DCD liver allografts that we otherwise would have been hesitant to use with SCS alone. Whether NRP truly improves outcomes compared with other retrieval and preservation techniques is unclear, given the lack of randomized controlled trials.

In conclusion, implementation of a TA-NRP program by abdominal transplant surgeons is feasible, facilitates the pursuit of abdominal-only DCD offers, and yields a decrease in the rate of IC compared with SCS in a real-world setting. As NRP proved to be a useful method to assess DCD livers via clinical and biochemical testing that is not traditionally available with SCS and improves recipient outcomes, these data support the ongoing development of NRP protocols for DCD LT.

## ACKNOWLEDGMENTS

The authors appreciate the efforts of Brooke Atkinson, RN, BSN, CMSRN, and Christina Nguyen-Newman of the UCH Transplant Quality team in managing NRP program operations and assisting with data collection. They value the UCH Division of Cardiothoracic Surgery and UCH perfusionists in helping learn the technical aspects of TA-NRP. They are thankful for the support of the local OPO, Donor Alliance.

## Supplementary Material


