# Supplementary material for: Thoracoabdominal Normothermic Regional Perfusion: Real-world Experience and Outcomes of DCD Liver Transplantation
Source: Transplant Direct. 2025 Feb 28;11(3):e1767. doi: 10.1097/TXD.0000000000001767 (PMC11875611; doi:10.1097/TXD.0000000000001767)
Supplement: Supplementary file 1 [file txd-11-e1767-s001.pdf]

Table S1. Case Review of All-Cause Recipient Mortality: Donation after Circulatory Death Normothermic Regional Perfusion Liver Transplantation

NRP Case Number	Reason for Graft Loss	Lab Na- MELD	Etiology of liver disease	DRI*	UK DCD Risk Score**	POD Graft Loss	Procurement Note	Hospital Course	Outcome
3	Patient death with a functioning graft in the setting of an aspiration event at rehab	20	NASH, HCC	1.43	8	132	NRP done by OSH; no microscopic or biochemical assessment on NRP	Discharged to rehabilitation facility with functioning graft	Cardiopulmonary arrest from aspiration event at rehabilitation facility
7	Patient death in the setting of acute liver failure from multi-organ sepsis	23	EtOH	2.11	3	100	NRP allograft biopsy only notable for 5% macrosteatosis; reassuring biochemical NRP parameters	Good graft function at discharge on POD 13	Readmitted POD 93 for multiorgan sepsis leading to acute liver failure and AKI requiring RRT. Autopsy did not identify ischemic cholangiopathy
28	PNF	20	NASH	2.66	3	42	NRP allograft biopsy only notable for 10% macrosteatosis; reassuring biochemical NRP parameters; macroscopic assessment noted allograft initially felt somewhat firm	Retransplanted with DBD	Retransplanted on POD 42 with rejection, persistent AKI requiring RRT, recurrent pleural effusions, large volume ascites, and allograft failure. Discharged to home after re-transplant with a DBD allograft
30	IC	17	PSC, EtOH	1.43	3	24	NRP done by OSH; no microscopic or biochemical assessment on NRP	Retransplanted with DBD	Initially discharged with good graft function. Re-admitted on POD 21 with acute liver failure with patent vasculature. Discharged to home after re-transplant via DBD allograft. Explant of DCD-NRP allograft demonstrated 60-70% lobular necrosis.
74	PNF	17	NASH	2.06	0	2	NRP allograft biopsy only notable for 20% macrosteatosis; reassuring biochemical NRP parameters. Macroscopic assessment noted allograft did not produce bile until end of NRP	Retransplanted with DCD TA-NRP	Discharged to home after re-transplant with a DCD TA-NRP allograft

DCD, donation after circulatory death; NRP, normothermic regional perfusion; LT, liver transplant; Na-MELD, Sodium Model for End Stage Liver Disease score calculated at time or organ offer; DRI, Donor Risk Index; UK, United Kingdom; POD, post-operative day; NASH, nonalcoholic steatohepatitis; EtOH, alcoholic liver disease; PSC, primary sclerosing cholangitis; OSH, outside hospital; AKI, acute kidney injury; RRT, renal replacement therapy; DBD, donation after brain death; TA, thoracoabdominal.
* Feng et al. Am J Transplant. 2006;6:783-790.
** Schlegel et al. J Hepatol. 2018;68:456-464.

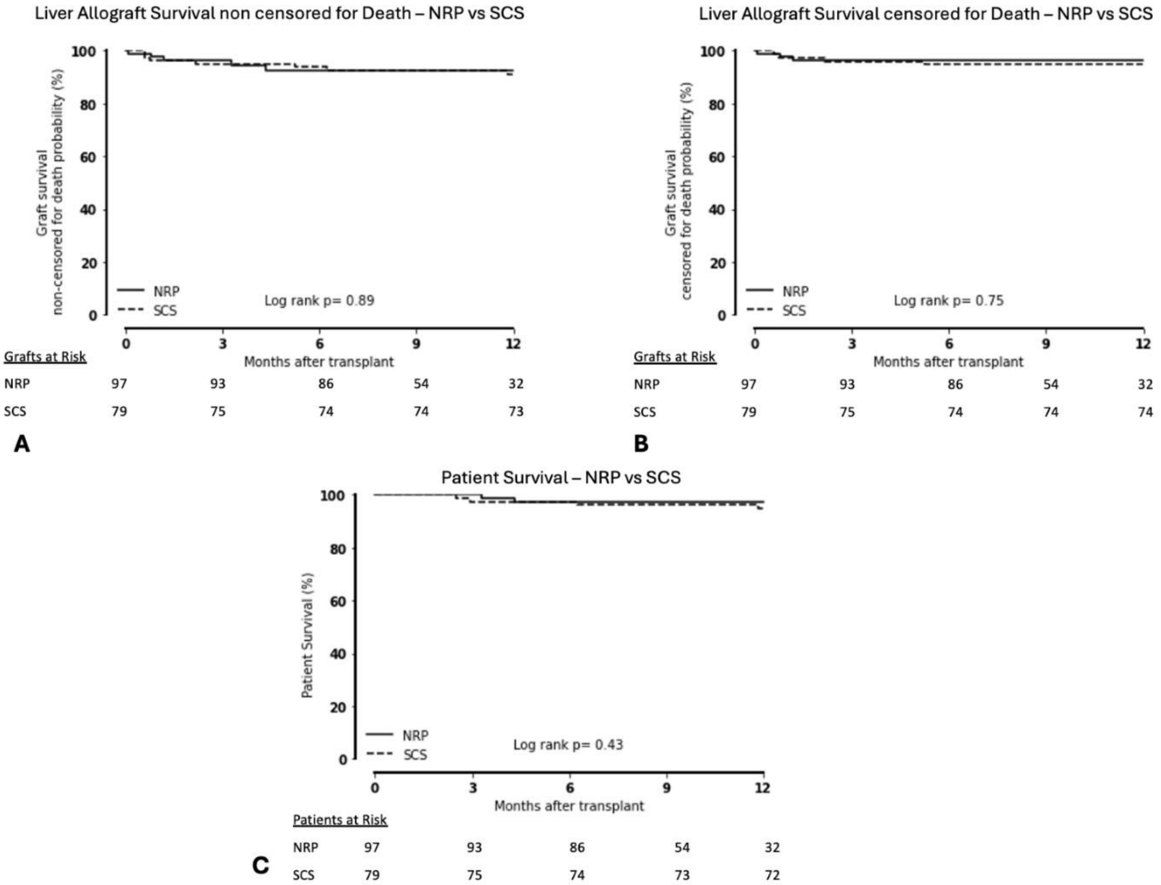

Figure S1. Liver Allograft and Patient Survival following Donation after Circulatory Death Liver Transplant: Normothermic Regional Perfusion vs Static Cold Storage
A) Kaplan-Meier graft survival, non-censored for death was not different between NRP and SCS (93% vs 91%, p=0.89)
B) Kaplan-Meier graft survival, censored for death was not different between NRP and SCS (96% vs 95%, p=0.75)
C) Kaplan-Meier patient survival was not different between NRP and SCS (97% vs 95%, p=0.43)

NRP, normothermic regional perfusion; SCS, static cold storage.

UNOS ID: Date of Cold Perfusion X Clamp: OPO:
Procurement Hospital: In Situ Assessment Surgeon: V: 12/12/2023

COLORADO NORMOTHERMIC REGIONAL PERFUSION IN SITU ASSESMENT: ABDOMINAL

Event	Date/Time	Event to on pump (min)	*Functional Warm Ischemia Guidelines*		Minutes	**Case Comments**
Extubation			*ASTS*	SBP<50 or O2<70 to on pump		
SBP <80			*ILTS*	MAP<60 or O2<80 to on pump		
SBP <50			*Liver Visual Assessment*			
MAP <60			Estimated Fat %	Soft/ Firm		
O2 <80			Procurement Trauma			
O2 <70			Liver Biopsy Y/N			
1st Declaration			Micro			
2nd Declaration			Macro			
Incision			Fibrosis			
On pump		--	Necrosis			
X Clamp		--	Other			

Parameters to be collected at initiation of NRP and in 15 minute intervals therafter. Record cummulative bile production if possible.
Target Cardiac Index for NRP: Target Circuit Flow for NRP:

NRP Paremeter	**PERFUSION**					**HEPATOCYTE**			**CHOLANGIOCYTE**			
Date/Time	Med/Blood/Fluid/ Circuit Issue	Circuit Flow	Cardiac Index	Serum pH	Base Deficit	Lactate	AST	ALT	Bile pH	Bile Glucose	Bile HCO3-	Bile Production

**Kidneys**	Accepting Center	Right Kidney:	Left Kidney:	**Pancreas**	Accepting Center:
Quality	Right Kidney Soft/Firm		Left Kidney Soft/Firm	Quality	Soft/Firm
UOP mL on NRP	Hour 1:	Hour 2: Hour 3:	Hour 4:		Pink Y/N
					Fatty Y/N

Figure S2. Colorado Normothermic Regional Perfusion in Situ Assessment Form: Abdominal Organs
The Colorado Normothermic Regional Perfusion *In Situ* Assessment Form is used by the procuring team at our institution to record and communicate mission critical variables during procurement.

SBP, systolic blood pressure; MAP, mean arterial pressure; X Clamp, cold perfusion aortic cross clamp; ASTS, American Society of Transplant Surgeons; ILTS, International Society of Liver Transplantation; Micro, microsteatosis; Macro, macrosteatosis; NRP, normothermic regional perfusion; AST, aspartate transferase; ALT, alanine transaminase; UOP, urine output.

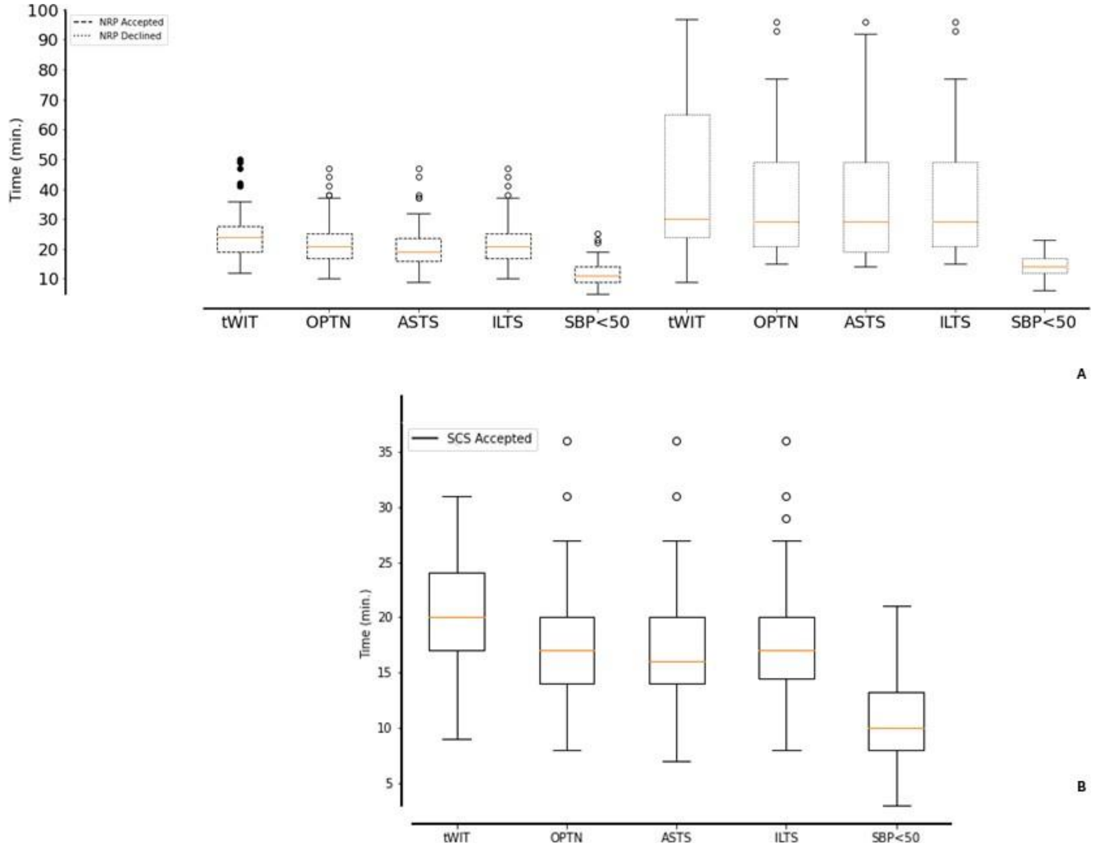

Figure S3. Warm Ischemia Times for Accepted and Declined Donation after Circulatory Death Normothermic Regional Perfusion Livers

A) Functional Warm Ischemia Time for Accepted and Declined Donation after Circulatory Death Normothermic Regional Perfusion Livers

B) Functional Warm Ischemia Time for Accepted Donation after Circulatory Death Static Cold Storage Livers

tWIT-total warm ischemia time; OPTN - fWIT SBP < 80 mmHg or O_2 Saturation < 80%; ASTS- fWIT SBP < 50 mmHg or O_2 Saturation < 70%; ILTS- fWIT SBP < 60 mmHg or O_2 Saturation < 80%; SBP<50 - fWIT SBP < 50 mmHg.

NRP, normothermic regional perfusion; tWIT, total warm ischemia time; fWIT, functional warm ischemia time; OPTN, Organ Procurement and Transplantation Network; ASTS, American Society of Transplant Surgeons; ILTS, International Liver Transplantation Society; SCS, static cold storage; SBP, systolic blood pressure.

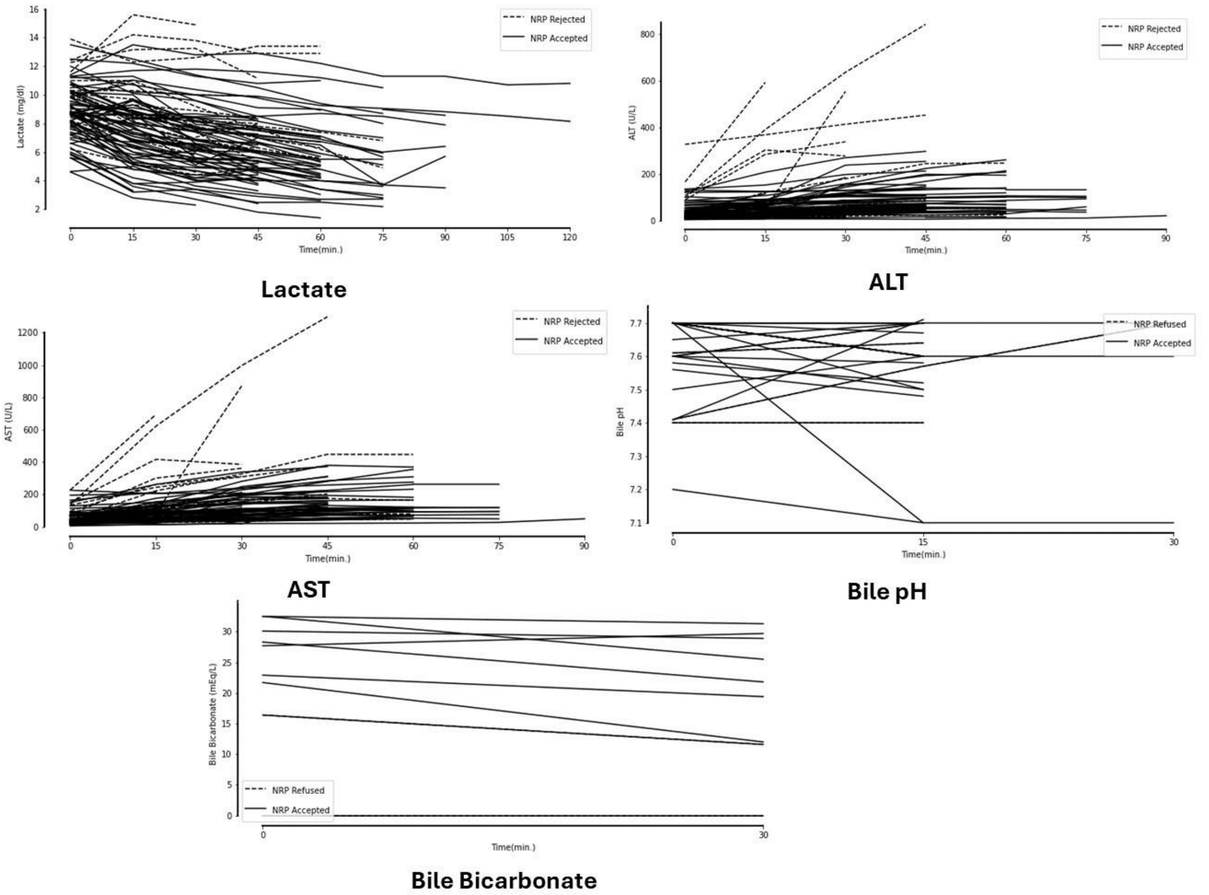

Figure S4. Values of Perfusion, Hepatocellular, and Cholangiocellular Parameters during DCD NRP

Biochemical assessments during NRP included labs to reflect perfusion, hepatocellular, and cholangiocellular parameters.

NRP, normothermic regional perfusion; ALT, alanine aminotransferase; AST, aspartate transaminase
